# A cluster randomised school-based lifestyle intervention programme for the prevention of childhood obesity and related early cardiovascular disease (JuvenTUM 3)

**DOI:** 10.1186/1471-2458-11-258

**Published:** 2011-04-22

**Authors:** Monika Siegrist, Henner Hanssen, Christoph Lammel, Bernhard Haller, Martin Halle

**Affiliations:** 1 Department of Prevention, Rehabilitation and Sports Medicine, Technische Universitaet Muenchen, Klinikum rechts der Isar, Munich, Germany; 2 Division of Sports Medicine, Institute of Exercise and Health Sciences, University of Basel, Switzerland; 3 Institute of Medical Statistics and Epidemiology, Technische Universitaet Muenchen, Klinikum rechts der Isar, Munich, Germany

**Keywords:** childhood obesity, school-based intervention, cardiovascular risk, arterial stiffness, retinal microcirculation

## Abstract

**Background:**

Childhood obesity is not only associated with adult obesity but also with increased risk of adult onset of type 2 diabetes and subsequent coronary heart disease. The potential effects of school-based health intervention programmes on cardiovascular risk and surrogate markers are unclear, as only few studies have attempted to investigate a complete risk profile including a detailed laboratory analysis or micro- and macrovascular function. In this study a comprehensive school-based randomized intervention programme will be investigated in 10-14-year old children addressing the influence of lifestyle intervention on inactivity, cardiometabolic risk factors and early signs of vascular disease.

**Methods/Design:**

15 secondary schools in Southern Germany are randomly assigned to intervention or control schools. Children in the fifth grade (10-11 years) will be observed over four years. The study combines a school-based with a home-based approach, aiming at children, teachers and parents. The main components are weekly lifestyle-lessons for children, taught by regular classroom teachers to increase physical activity in- and outside of school, to improve eating patterns at school and at home, to reduce media consumption and to amplify well-being. In 4-6 annual meetings, teachers receive information about health-related topics with worksheets for children and supporting equipment, accounting for school-specific needs and strategies. Parents' trainings are provided on a regular basis.

All examinations are performed at the beginning and at the end of every school year. Anthropometry includes measurements of BMI, waist and upper arm circumferences, skinfold thickness as well as peripheral blood pressure. Blood sampling includes lipid parameters, insulin, glucose, hsCRP, adiponectin, and IL-6 as well as testosteron and estrogen to determine maturation status. Vascular function is non-invasively assessed by measuring arterial stiffness in large arteries using a sphygmograph and by analysing arteriolar and venular diameters in the retinal microcirculation using a non-mydriatric vessel analyser. A questionnaire is filled out to determine daily physical activity, motivational factors, dietary habits, quality of life (KINDL-R) and socio-economic data. Physical fitness is assessed by a six-item test battery.

**Discussion:**

Our study aims to provide a feasible long-term intervention strategy to re-establish childhood health and to prevent obesity-related cardiovascular dysfunction in children.

**Trial Registration:**

NCT00988754

## Background

### Cardiovascular Disease Epidemiology

According to the World Health Organisation, cardiovascular disease (CVD) is the leading cause of mortality worldwide and is responsible for 30% of all global deaths [1]. In adults, a high body mass index is the main reason for increased mortality [2]. Adult and childhood overweight and obesity are often related to manifestation of metabolic diseases such as type 2 diabetes, hypertension and hyperlipidemia [3-6]. Overweight and obesity can often be tracked from childhood to adulthood [7,8] and childhood obesity has been associated with premature death [9,10] and adult coronary heart disease [11,12].

### Eating Pattern, Physical Activity and Mortality

The worldwide increase in obesity is related to changes in eating patterns and reduced physical activity. Caloric-dense foods with high satured fat and refined carbohydrates have replaced traditional diets with vegetables, fruits and whole grain products. The increase in fast-food restaurants [13] and "eating out" is associated with a higher risk to become overweight [14]. Sugar-sweetened beverages induce weight gain and increase the incidence of type 2 diabetes [15]. Unhealthy eating patterns are often combined with less physical activity.

It is estimated that 60% of the population all over the world is inactive. Physical inactivity is estimated to be the main cause for approximately 21-25% of breast and colon cancers, 27% of diabetes and approximately 30% of the ischaemic heart disease burden [16]. Physical inactivity and low physical fitness is associated with a higher risk for hypertension and stroke [17] and type 2 diabetes [18], independent of overweight and obesity.

Results of the German Health Interview and Examination Survey for Children and Adolescents (KiGGS) have shown that only 28% of boys and 17% of girls reach the current recommendation of 60 minutes or more of physical activity each day (CDC 2010, WHO 2010). Low activity is particularly prevalent in girls, as well as in children with low socio-economic status or ethnic background [19]. In the United States only 15% of the children go or bike to school (CDC 2010). In UK, the average annual walking distances has declined by 24% and the annual distance cycled by 31% from 1985 to 1995 in adolescents aged 15-19 years [20].

These lifestyle changes lead to obesity, chronic diseases and premature mortality [21].

### Childhood Obesity, Physical Activity and Inflammation

The association between c-reactive protein (CRP) and future coronary events has been demonstrated for adults [22]. CRP is a sensitive marker of inflammation and it plays a causal role in the process of inflammation [23]. In healthy prepubertal children an association between CRP, fasting insulin, dyslipidemia, blood pressure and adiposity has been found [24]. In the Cardiovascular Risk in Young Finns Study, high CRP levels were one of the determinants in youth for the incidence of adult metabolic syndrome [25]. In obese and normal weight adolescents, regular aerobic exercise has been shown to reduce CRP concentrations [26,27].

Also interleukin-6 (IL-6) plays a key role in the pathogenesis of CVD [28]. It affects platelet reactivity as well as endothelial function [29]. IL-6 is secreted to a large extend in adipocytes of visceral fat and is therefore elevated in obese individuals [30,31]. In addition, IL-6 seems to be negatively associated with physical activity [32] and regular exercise has the potential to decrease IL-6 serum levels [27].

The adiponectin, an adipokine, is reduced in obesity and type 2 diabetes. Low plasma adiponectin levels are a sign of decreased insulin sensitivity, adipocyte dysfunction and an important link to the development of vascular disease [33-35]. In children, adiponectin is also inversely correlated with BMI, fat mass and fasting insulin [31]. The effects of physical activity on adiponectin levels in children, however, are still unclear.

In summary, physical activity and physical fitness seem to be protective against low grade inflammation, but more longitudinal research is needed to clarify the association between physical activity, physical fitness, obesity and low grade inflammation in children [36].

### Childhood Obesity, Physical Activity and Atherosclerosis

Physical inactivity has been shown to play a key role in the development of obesity-related atherosclerotic cardiovascular disease and may represent an important link between obesity, inflammation, insulin resistance and early atherosclerosis in adults [37]. However, the association among children is less clear and has only been examined by a handful of studies [38]. Increased carotid intima-media thickness (IMT) and impaired flow-mediated vasodilation (FMD) have previously been documented in young obese subjects which can be restored after a six-months exercise programme [26]. Furthermore, in obese children, a higher arterial wall stiffness has previously been described [39]. Pulse wave velocity (PWV) is a validated index of arterial stiffness which can easily be examined by non-invasive applanation tonometry. In healthy prepubescent children, increased PWV is associated with obesity and decreased cardiorespiratory fitness [40].

The retinal microcirculation can be analysed non-invasively, which offers a unique opportunity to examine the effects of obesity on small brain vessels. In adults, obesity has been associated with a wider retinal venular diameter and a lower arterio-venous ratio (AVR). In several large adult cohort studies, AVR has been shown to be a valid index for an increased risk of hypertension, stroke and higher cardiovascular mortality [41,42]. Similar to findings in adults, childhood obesity is associated with retinal venular dilatation and lower AVR [43], and higher childhood blood pressure has been linked to retinal arteriolar narrowing [44]. The investigation of early macro- and microvascular impairments in childhood obesity are important measures to define obesity-related early signs of vascular disease. No study to date has examined the effects of a lifestyle intervention on the retinal microcirculation in children. Therefore, it is of great importance to elucidate whether early vascular alterations in children can be restored by a school-based intervention programme.

### Media Use, Physical Activity and Health

Over the last twenty years, the amount of time children spend in front of television, computer and video games has increased and serious negative health effects such as violent behaviour, substance abuse (alcohol, smoking), decreased school performance, poor body image and obesity become more apparent [45]. Television affects food consumption of children [46] and displaces physical activity [47]. Reducing media use has been shown to be an effective measure in preventing obesity [48,49] and improving educational achievements [50]. Moreover, increased physical activity has been demonstrated to combine health benefits with reduced substance abuse [51].

### Effects of School-based Physical Activity Interventions

The school environment has great potentials to introduce and encourage a healthy lifestyle in children across all socio-economic and ethnic borders. However, it becomes more and more evident that the school-intervention approach needs to be combined with a family-based health education [52]. Several lifestyle intervention programmes have been conducted in school settings, but the results have been inconsistent and have primarily focused on basic measures of obesity such as BMI or skinfold thickness [53-55]. Although anthropometric end points are fundamental, more attention needs to be drawn to obesity-related cardiovascular risk factors such as lipid profiles, adipocytokines, inflammation and insulin metabolism. Some more recent studies have demonstrated the beneficial effects of a school-based intervention on serum lipids and inflammatory markers in obese children [56]. As childhood obesity is associated with signs of early atherosclerosis, school-based interventions should be validated with a long follow-up regarding improvement of behaviour (diet, exercise), body composition, fitness, and early cardiovascular risk profile.

## Methods/Design

### Objectives

The study aims to implement a comprehensive randomized controlled school- and family-based lifestyle-intervention trial (RCT) in secondary schools to analyse and improve cardiometabolic risk factors and vascular function in large and small vessels of children aged 10 to 11 years over a period of four years by increasing physical activity and physical fitness, psychological well-being, and the motivation to exercise.

### Primary Objectives

1. Primary outcomes of the intervention were defined as an increase in number of days with physical activity >60 min/day (in- and outside of school).

### Further Objectives are

2. Prevention of early macro- and microvascular impairments (arterial stiffness and retinal vessel diameter).

3. Reduction of cardiometabolic risk factors (Triglycerides, LDL-cholesterol, HDL-cholesterol, glucose, hsCRP, IL-6, adiponectin).

4. Reduction of the prevalence of obesity (BMI, waist circumferences, total skinfold thickness).

5. Improvement in motor abilities and physical fitness (six-item test battery).

6. Normalisation in systolic and diastolic blood pressure.

7. Increase in health-related quality of life (HRQoL) measured by a child questionnaire (KINDL-R).

### Secondary Objectives

To analyse the correlation between arterial stiffness and retinal vessel diameter with childhood obesity and interventional improvements of cardiovascular risk factors. In addition, the association between arteriel stiffness, retinal vessel diameter and lifestyle factors (physical activity, eating patterns, smoking are elucidated.

### Study Groups/Recruitment

**Figure 1:**
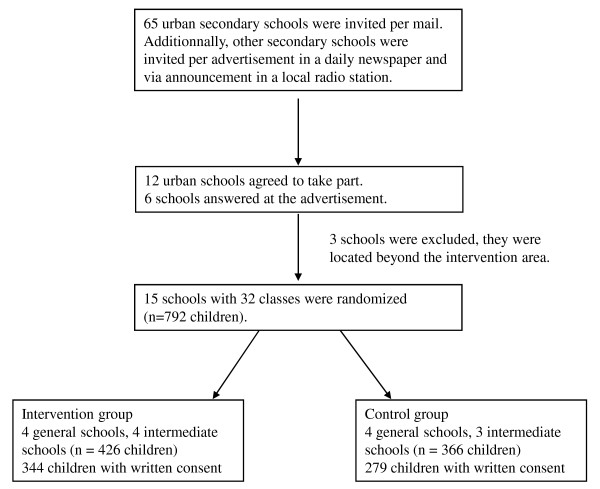
**Flow of School Classes**.

Figure 1 shows the flow diagram of the recruitment process. All secondary schools in the greater district of a city in Southern Germany were asked to take part by written invitations (65 schools), an advertisement in a local newspaper and via local radio announcements. Recruitment of participating schools was based on the willingness to take part over four years and to be randomized either to an intervention school with a lifestyle intervention programme or a control school. 12 urban schools agreed to take part in response to the individual invitations and interviews, 6 schools answered the advertisement in the newspaper. From these, 3 schools were excluded because they were located outside of the study area (distance >30 km). In all, 15 schools with 32 classes agreed to take part and were randomized. The children of 7 secondary general schools and 8 intermediate schools gave written consent from their parents. 12 schools are located in the city area and three schools are located in communities outside the greater city area. The study protocol was approved by the ethics board of the faculty of medicine of the Technische Universitaet Muenchen. The study takes into account the principles set out in the Helsinki declaration (Seoul, 2008) (2119/08).

### Statistical Analysis

Fifteen schools with about 700 pupils attending fifth grade agreed to participate in the study and were randomly assigned to either intervention (n = 8 schools) or control group (n = 7 schools). Aim of the study is to increase the number of days per week a child is active, defined as days with physical activity of more than 60 minutes. This outcome is assessed in a standardized questionnaire at the beginning and at the end of the study. The primary analysis will be a comparison of the proportion of children with an increase in the number of active days per week between both study groups considering the effect of clustering.

Due to the small number of cluster (n = 15) methods analysing the data on the individual level and accounting for the intracluster correlation (as e.g. GEE [57,58]) or adjusted chi-square test [57] are not applicable [59]. Therefore the primary endpoint will be analysed on the cluster level. Mean proportions of children with increased activity assessed at school level will be compared between control and intervention schools as proposed by Donner and Klar [57] for the analysis of binary data in cluster randomized trials with a small number of clusters. A two-sample t-test will be performed to compare both groups. The t-test was shown to give valid results in such settings, even when assumptions (normality, equality of variances) are moderately violated [60]. Since drop-outs of children will be mainly caused by school changes and therefore are assumed to be independent of the outcome, a complete case analysis will be performed to investigate the primary endpoint. Yearly examinations will be considered to investigate the pattern of missing values. If the assumption of random drop-outs does not hold, adequate imputation methods will be used. The primary analysis will be performed on a two-sided level of significance of α = 0.05.

All secondary endpoints will be analysed on the cluster level. 95% confidence intervals will be presented for relevant measures. For binary data, means of school level proportions, for quantitative data means of school level means will be compared using two-sample t-tests. All secondary endpoints will be analysed in an explorative manner on a two-sided level of significance of α = 0.05, so no adjustment for multiple comparisons will be conducted.

### Power Considerations

In a similar trial in younger children (JUVENTUM I, currently under review) a standard deviation for the school level proportion of children increasing their activity of 9 percentage points was observed in both groups. Assuming this standard deviation, the study is sufficiently powered (over 80%) to detect a difference between control and intervention group of 15 percentage points, translating to an effect size of 1.67, on a two-sided level of significance of α = 0.05.

### Intervention

This lifestyle intervention programme aims to increase physical activity in schools and at home by the following means: regular physical exercise in sports lessons, additional school-based exercise such as active breaks during regular lessons and active school breaks, improving playing facilities at school, increasing sports activities at home. Furthermore, the programme intends to improve eating patterns (less sweetened drinks, regular healthy breakfast, healthy meals at school, daily fruits and vegetables), to reduce media use, to prevent substance abuse and to improve well-being.

This comprehensive prevention programme was planned according to the social cognitive theory [61] with the following components: information for children and teachers about health risks and benefits of different lifestyle habits in lifestyle-lessons, regular information and lessons for parents, little extra homework and tasks to encourage social and self-management skills, enable all participants to translate the knowledge and skills into effective preventive practices, strengthen children as well as teachers to control difficulties and setbacks in regular discussions, create social supports for individual health behaviour changes (structural changes, development of a "healthy school").

Children obtain weekly health education lessons from their regular teachers to increase physical activity in and outside of school and to improve the health behaviour (healthy food, no drugs) as well as the general well-being of children. Each topic will be discussed over a period to 4-6 weeks. Children receive worksheets, specific homework or practical instructions to transfer the healthy lifestyle in daily activities.

The teachers will be trained regularly in 4-6 annual meetings to perform the lifestyle-lessons with their pupils. The contents of the lifestyle-lessons are explained and discussed and additional activities in schools are planned (arrangements in classrooms and playgrounds, implementation of healthy meals at school, healthy lifestyle for teachers and other employees at school).

**Figure 2:**
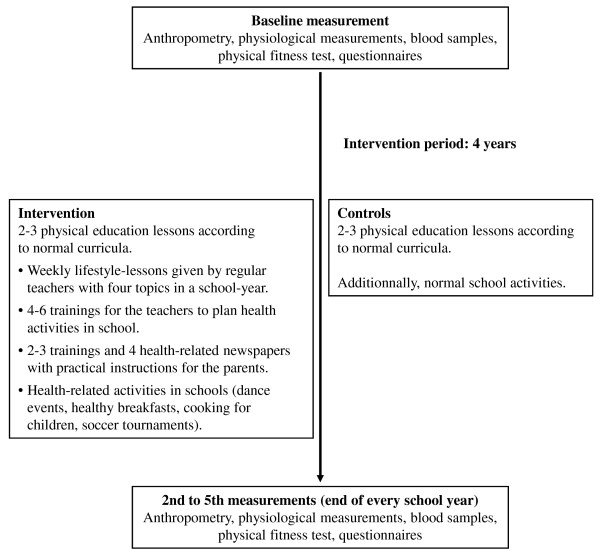
**Content and Timetable of Intervention**.

Parents will be informed about the programme and the health-related topics of the lifestyle-lessons by regular newspapers (4x/year). They are invited to trainings (2-3x/year), which include cooking with their children, media use and hands-on-sessions for a more active lifestyle in the family. Schools' health-related practices are analysed and included in the approach of the intervention. Control schools are asked to continue their usual school curriculum (Figure 2).

### Course of Measurements

All measurements and fitness tests are performed on a yearly basis in every school over a period of four years (Table 1). The examinations are to be carried out individually by trained staff according to standardized operating procedures during classroom lessons in three separated rooms inside the schools. The physical fitness tests are conducted by trained staff in the physical exercise lessons in the sports halls on a second examination day. The questionnaires are filled out in class.

**Table 1:** Data Collection

Personal Level	Data
Anthropometry	Name, date of birth, sex, type of school, medication, diseases, height, weight, waist and upper arm circumferences, skinfold thickness
Physiological measurements	Blood pressure, retinal vascular caliber, arterial stiffness
Blood samples	Triglyzerides, HDL- and LDL-cholesterol, insulin, fasting glucose, adiponectin, IL-6, hsCRP, testosteron, estrogen
Physical fitness	Six-item test battery
Physical activity	Self reported activity level in school and at home
Eating patterns	Food frequency questionnaire
Substance abuse	Smoking and consumption of alcohol
Life quality	KINDL-R-Questionnaire
Sedentary behaviour	Questionnaire concerning sedentary time (school, homework, media use)
Socio-economic index	Questionnaire concerning school level parents, native country of the parents and child, nationality children and parents, first language child
**School Level Data**	
Questionnaire	Compliance to the intervention programme, school-specific needs, health-related activities in school (only in intervention schools)

Both children and parents are informed (in German and Turkish) that they may withdraw from the programme at any point and that all information collected is confidential. Only data from children with written consent is included in the anonymous data analysis. Teachers, children and parents are informed about their programme status and will be aware if they are in an intervention or control school.

### Anthropometrics

All clinical examinations are conducted by trained medical staff according to standardized procedures. Height and weight are measured at the beginning of the programme and annually at the end of a school year with minimal clothing without shoes. Weight is measured to the nearest 0.1 kg by using a calibrated balance scale. Height is measured to the nearest 0.1 cm by a rigid stadiometer. Body mass index (BMI) may then be calculated as weight in kilogramms divided by the square of height in meters. The BMI-SDS (BMI-standard deviation score) is determined using the LMS-method [62]. Reference data from German children are used [63]. Children with a BMI lower than the 10^th ^percentile are classified as underweight, between the 10^th ^to 90^th ^percentile as normal, between the 90^th ^and 97^th ^percentile as overweight, and above the 97^th ^percentile as obese. The pubertal development stage is being determined by estrogen and testosteron levels. Waist circumference is measured on bare skin in a standing relaxed position in a horizontal line just above the navel, the upper arm circumference is examined half way between the olecranon process of the ulna and the acromion process of the scapula with a non-stretchable tape measure. Skinfold thickness as a surrogate of subcutaneous fat is measured at four sites (m. bizeps, m. triceps, subscapular and supra-iliacal) according to the guidelines of the manufacturer. Blood pressure is measured by using a validated protocol [64] at the right brachial artery in the fossa cubitalis after the subjects rest for 5 min in a supine position. Measures of systolic and diastolic blood pressures and the cuff sizes are to be recorded.

### Blood Sampling and Analysis

Blood sampling is performed in fasting status. Blood samples are taken by venipuncture of an antecubital vein in either sitting or lying position after an overnight fast, using vacuum tubes. Blood samples are collected by qualified medical staff. Breakfast is to be provided after blood sampling to all children in the classroom. Blood samples will immediately be transported to the laboratory to be analysed. Samples will be analysed for triglyzerides, LDL- and HDL-cholesterol, fasting glucose, insulin, adiponectin, IL-6, hsCRP (high sensitive CRP), testosteron and estrogen, respectively.

### Arterial Stiffness and Retinal Vascular Caliber

Arterial stiffness in children is assessed by use of a sphygmograph (SphygmoCor SCOR-Px, AtCor Medical Pty. Ltd., Sydney Australia) following a standardized protocol validated in adults to detect early macrovascular changes. The system uses an applanation tonometry device connected to an electronic module to non-invasively record and analyse pulse wave forms of the radial artery in children after 5 minutes of rest in a lying position. From these measurements, the calibrated ascending aortic blood pressure waveforms can be estimated. Accordingly, central systolic and diastolic blood pressure, pulse pressure, augmentation pressure and the augmentation index (AiX@HR75) are computed as described elsewhere [65]. For each measurement, an average of ten pulse waves is taken to calculate the above parameters. For each child at least two valid, quality-controlled measurements (operator index ≥75%) are recorded and averaged.

The diameters of retinal arterioles and venules are measured using a Static Retinal Vessel Analyser (SVA-T, Imedos Systems UG, Jena, Germany) to detect early microvascular changes. The system allows non-invasive online measurement of the diameter of retinal vessels without mydriasis. It consists of a fundus camera and an advanced image processing unit [66]. For static analysis, two valid images are taken from the retina of the right eye with the optic disc in the center. Retinal arterioles and venules are identified using special analysing software identifying retinal vessels in ring-zones (Vesselmap 2, Visualis, Imedos Systems UG). All retinal arterioles and venules are differentiated by the examiner in the outer ring zone and measured by the automated software. Diameters are calculated to central retinal arteriolar and venular equivalents (CRAE, CRVE), using the Parr-Hubbard formula described elsewhere [66]. The CRAE and CRVE are used to calculate the arteriolar-to-venular ratio (AVR), taking the mean of both measurements.

### Physical Fitness

Physical fitness is measured by the Munich fitness test. This standardized test includes 6 items (step test, goal throwing, stand-and-reach, jump-and-reach, flexed arm hanging, ball bouncing) designed to evaluate cardiopulmonary fitness, coordination, muscle strength, and flexibility in children. For each item, participants receive gender- and age-specific t-scores between 30 and 70 points. The sum of the 6 items divided by the number of items yields the total t-score [67].

### Questionnaires

Standardized questionnaires were used to obtain demographic variables (native country and nationality of the parents and children, first language, school duration, graduation and working position of the parents, number of family members).

Physical activity is measured by a validated questionnaire concerning the amount of moderate-to-vigorous physical activity [68]. Further items are duration, intensity, and frequency in leisure time, school and sports clubs activities as well as motivation to be physically active [69]. Additionnally, the amount of physical activities of parents, siblings, and peers is asked.

For the assessment of dietary intake, a food frequency recall is used according to a questionnaire which was used recently in a large german survey [70]. The quality of life is determined by the 24-item 5-point Likert scale KINDL-R-questionnaire [71] with subscales for physical well-being, emotional well-being, self-esteem, family, friends, and everyday functioning. Additionnally, smoking patterns and the consumption of alcohol are asked.

### Feedback Questionnaire

At the end of every school year the teachers document the number and contents of the lifestyle-lessons as well as all other health-related activities at school. Additionnally, topics for the next school year may be proposed.

## Discussion

Previous reviews on school-based childhood obesity prevention programmes have so far been unable to show clear evidence for the efficacy of intervention programmes, limited by the small number of published studies and by methodological concerns [54]. Most interventions did not improve BMI. But it is known that physical activity can increase lean muscle mass and decrease fat mass with no changes in BMI [53]. Additionally, physical activity has direct effects on metabolic function and cardiovascular risks [72]. Relevant health effects such as normalisation of lipid profiles and inflammatory markers or, most importantly, improvements of vascular end organ damage are not detected focusing on anthropometric parameters only.

In our study, we aim to implement an integrated approach by combining the analysis of anthropometric data with cardiometabolic risk factors and measures of vascular function. Our objectives are to thereby detect children with increased cardiovascular risk and signs of early vascular impairments. We further aim to demonstrate that the comprehensive school-based intervention programme has the potential to restore vascular changes in obese children.

Pulse wave and retinal vessel analysis are key elements of the study design and both are non-invasively applicable in children. They help to visualize and communicate direct health benefits of the longitudinal intervention to participating children, parents and teachers.

Numerous investigations have demonstrated a decrease in physical activity and physical fitness in children. The school environment can play an important role in increasing the physical activity of children. The way to school is an important part of daily activity and facilitates children to reach the current recommendations [73]. Therefore, active commuting should be supported by schools and parents. An increasing number of children in Germany spend the whole day at school. Schools should therefore aim to offer physical activity practice in regular lessons, to carry out sufficient physical exercise lessons with qualified teachers, as well as to provide sufficient physical activity proposals during breaks and in the afternoon. Beyond these school-based efforts, the prevention programme will encourage family-based physical activities and reinforce the cooperation between schools and sports clubs. In addition, the programme educates children and parents on healthy food and eating patterns in order to improve their skills to achieve a healthy lifestyle. Advice is given to the schools' administrations to implement healthy food in school vending machines and school meals.

Over the last decade, the growing influence of internet and social networks as well as the increase of media use has augmented enormously, especially in children. School-based prevention programmes therefore need to include media-competence-training to prevent a further decline in physical activity, negative effects on school performance or well-being.

This health-promoting prevention programme is based on the following components: information for children, teachers and parents about important health-related topics, encouragement to translate knowledge and skills into daily practice, discuss difficulties and setbacks that arise in everyday life. The programme aims to target and improve the personal responsibility of the children, strengthened by the healthy attitudes of teachers and parents likewise. The comprehension of personal responsibilities is an important focus of the programme. In school children, puberty represents a point of time where unhealthy lifestyle often originates (smoking, alcohol abuse, excessive media use, inactivity, psycho-social difficulties). Participating schools will be provided with worksheets for the children and health-related information for their parents, in order to facilitate the transfer from school to family routine. Weekly lifestyle-lessons over a period of four years aim to ensure sustainable health effects.

Our study has some limitations. First, all children, parents and teachers needed to be informed about the group allocation. The main coordinator of the study is also not blinded to the group assignments of the schools. However, the medical examiners are not aware of the group allocation of the participating children. This programme is conducted in 15 secondary schools and all assessments need to be integrated in the daily routine of the schools. On the basis of the number of participants at baseline (n = 792), physical activity can only be measured by questionnaire and not by accelerometers. Also, eating patterns are assessed by a food frequency questionnaire and not by weighed dietary records, which do not seem to be applicable to daily school routine.

The main strength of the study approach is the assessment of macro- and microvascular changes as an early functional marker of an increased cardiovascular risk in children with different body composition and levels of activity. In fact, this is the first school-based intervention study to include this integrative approach. The results of this study may help bridge the pathophysiological gap between childhood obesity and the reported associated increased risk of adult coronary heart disease and premature death [9,11,12,44].

We hypothesize that our intervention can easily be integrated in the daily routine of schools and that it proves to be an effective means to increase the physical activity levels of children in school and at home. We expect activity- and lifestyle-induced improvements of lipid profiles, low grade inflammation and metabolic risk factors as well as the normalisation of vascular impairments in children at risk. If the expected health benefits can be confirmed during the course of the programme, this interventional setting may have the potential to act as a model for future interventions and to emphasize the importance of detecting early vascular changes in children. Vascular end points may proof to be better predictors for the success of early life style interventions on childhood and future adult health.

## Competing interests

The authors declare that they have no competing interests.

## Authors' contributions

MS, HH and MH designed and wrote the original proposal. CL and BH were responsible for analysing and interpreting data. All authors have read and approved the final manuscript.

## Pre-publication history

The pre-publication history for this paper can be accessed here:

http://www.biomedcentral.com/1471-2458/11/258/prepub

## References

[B1] Bonow RO World Heart Day 2002: the international burden of cardiovascular disease: responding to the emerging global epidemic Circulation 2002 106 13 1602 1605 10.1161/01.CIR.0000035036.22612.2B 12270848

[B2] Berrington dGA Body-mass index and mortality among 1.46 million white adults N Engl J Med 2010 363 23 2211 2219 10.1056/NEJMoa1000367 21121834 PMC3066051

[B3] Ekelund U Prevalence and correlates of the metabolic syndrome in a population-based sample of European youth Am J Clin N 2009 89 90 96 10.3945/ajcn.2008.26649 19056570

[B4] Morrison JA Metabolic syndrome in childhood predicts adult metabolic syndrome and type 2 diabetes mellitus 25 to 30 years later J Pediatr 2008 152 2 201 206 10.1016/j.jpeds.2007.09.010 18206689

[B5] Srinivasan SR Adolescent overweight is associated with adult overweight and related multiple cardiovascular risk factors: the Bogalusa Heart Study Metabolism 1996 45 2 235 240 10.1016/S0026-0495(96)90060-8 8596496

[B6] Sun SS Childhood obesity predicts adult metabolic syndrome: the Fels Longitudinal Study J Pediatr 2008 152 2 191 200 10.1016/j.jpeds.2007.07.055 18206688 PMC3988700

[B7] Dietz WH Childhood weight affects adult morbidity and mortality J Nutr 1998 128 2 Suppl 411:S 414S 10.1093/jn/128.2.411S 9478038

[B8] Freedman DS Relation of body mass index and skinfold thicknesses to cardiovascular disease risk factors in children: the Bogalusa Heart Study Am J Clin Nutr 2009 90 1 210 216 10.3945/ajcn.2009.27525 19420092 PMC2697002

[B9] Franks PW Childhood obesity, other cardiovascular risk factors, and premature death N Engl J Med 2010 362 6 485 493 10.1056/NEJMoa0904130 20147714 PMC2958822

[B10] Reilly JJ Kelly J Long-term impact of overweight and obesity in childhood and adolescence on morbidity and premature mortality in adulthood: systematic review Int J Obes (Lond) 2010 in press 10.1038/ijo.2010.222 20975725

[B11] Baker JL Olsen LW Sorensen TI Childhood body-mass index and the risk of coronary heart disease in adulthood N Engl J Med 2007 357 23 2329 2337 10.1056/NEJMoa072515 18057335 PMC3062903

[B12] Bibbins-Domingo K Adolescent overweight and future adult coronary heart disease N Engl J Med 2007 357 23 2371 2379 10.1056/NEJMsa073166 18057339

[B13] Fraser LK The geography of Fast Food outlets: a review Int J Environ Res Public Health 2010 7 5 2290 2308 10.3390/ijerph7052290 20623025 PMC2898050

[B14] Naska A Eating out, weight and weight gain. A cross-sectional and prospective analysis in the context of the EPIC-PANACEA study Int J Obes (Lond) 2011 35 3 416 26 10.1038/ijo.2010.142 20661252

[B15] Schulze MB Sugar-sweetened beverages, weight gain, and incidence of type 2 diabetes in young and middle-aged women JAMA 2004 292 8 927 934 10.1001/jama.292.8.927 15328324

[B16] WHO Global Recommendations on Physical Activity for Health WHO Library 2010 ISBN 978 92 4 159 997 9 26180873

[B17] Goldstein LB Guidelines for the Primary Prevention of Stroke. A Guideline for Healthcare Professionals From the American Heart Association/American Stroke Association Stroke 2010 42 2 517 584 21127304 10.1161/STR.0b013e3181fcb238

[B18] Colberg SR Exercise and type 2 diabetes: the American College of Sports Medicine and the American Diabetes Association: joint position statement Diabetes Care 2010 33 12 e147 e167 10.2337/dc10-9990 21115758 PMC2992225

[B19] Lampert T Körperlich-sportliche Aktivität von Kindern und Jugendlichen in Deutschland. Ergebnisse des Kinder- und Jugendgesundheitssurveys (KIGGS) Bundesgesundheitsblatt - Gesundheitsforschung - Gesundheitsschutz 2007 50 5/6 634 642 17514447 10.1007/s00103-007-0224-8

[B20] DiGuiseppi C Li L Roberts I Influence of travel patterns on mortality from injury among teenagers in England and Wales, 1985-95: trend analysis BMJ 1998 316 7135 904 905 9552840 10.1136/bmj.316.7135.904 PMC28495

[B21] Cecchini M Tackling of unhealthy diets, physical inactivity, and obesity: health effects and cost-effectiveness Lancet 2010 376 9754 1775 1784 10.1016/S0140-6736(10)61514-0 21074255

[B22] Ridker PM High-sensitivity C-reactive protein and cardiovascular risk: rationale for screening and primary prevention Am J Cardiol 2003 92 4B 17K 22K 12948872 10.1016/s0002-9149(03)00774-4

[B23] Pasceri V Willerson JT Yeh ET Direct proinflammatory effect of C-reactive protein on human endothelial cells Circulation 2000 102 18 2165 2168 11056086 10.1161/01.cir.102.18.2165

[B24] Ford ES C-reactive protein and body mass index in children: findings from the Third National Health and Nutrition Examination Survey, 1988-1994 J Pediatr 2001 138 4 486 492 10.1067/mpd.2001.112898 11295710

[B25] Mattsson N Childhood predictors of the metabolic syndrome in adulthood. The Cardiovascular Risk in Young Finns Study Ann Med 2008 40 7 542 552 10.1080/07853890802307709 18728920

[B26] Meyer AA Improvement of early vascular changes and cardiovascular risk factors in obese children after a six-month exercise program J Am Coll Cardiol 2006 48 9 1865 1870 10.1016/j.jacc.2006.07.035 17084264

[B27] Rosenbaum M School-based intervention acutely improves insulin sensitivity and decreases inflammatory markers and body fatness in junior high school students J Clin Endocrinol Metab 2007 92 2 504 508 17090635 10.1210/jc.2006-1516

[B28] Van Snick J Interleukin-6: an overview Annu Rev Immunol 1990 8 253 278 10.1146/annurev.iy.08.040190.001345 2188664

[B29] Lindmark E Relationship between interleukin 6 and mortality in patients with unstable coronary artery disease: effects of an early invasive or noninvasive strategy JAMA 2001 286 17 2107 2113 10.1001/jama.286.17.2107 11694151

[B30] Fried SK Bunkin DA Greenberg AS Omental and subcutaneous adipose tissues of obese subjects release interleukin-6: depot difference and regulation by glucocorticoid J Clin Endocrinol Metab 1998 83 3 847 850 10.1210/jc.83.3.847 9506738

[B31] Nemet D Adipocytokines, body composition, and fitness in children Pediatr Res 2003 53 1 148 152 12508095 10.1203/00006450-200301000-00025

[B32] Platat C Relationships of physical activity with metabolic syndrome features and low-grade inflammation in adolescents Diabetologia 2006 49 9 2078 2085 10.1007/s00125-006-0320-6 16791618

[B33] Hopkins TA Adiponectin actions in the cardiovascular system Cardiovasc Res 2007 74 1 11 18 10.1016/j.cardiores.2006.10.009 17140553 PMC1858678

[B34] Kadowaki T Adiponectin and adiponectin receptors in obesity-linked insulin resistance Novartis Found Symp 2007 286 164 176 18269182 10.1002/9780470985571.ch15

[B35] Lindsay RS Adiponectin and development of type 2 diabetes in the Pima Indian population Lancet 2002 360 9326 57 58 10.1016/S0140-6736(02)09335-2 12114044

[B36] Thomas NE Williams DR Inflammatory factors, physical activity, and physical fitness in young people Scand J Med Sci Sports 2008 18 5 543 556 10.1111/j.1600-0838.2008.00824.x 18627553

[B37] Grundy SM Primary prevention of coronary heart disease: integrating risk assessment with intervention Circulation 1999 100 9 988 998 10468531 10.1161/01.cir.100.9.988

[B38] McGill HC McMahan CA Gidding SS Preventing heart disease in the 21st century: implications of the Pathobiological Determinants of Atherosclerosis in Youth (PDAY) study Circulation 2008 117 9 1216 1227 10.1161/CIRCULATIONAHA.107.717033 18316498

[B39] Tounian P Presence of increased stiffness of the common carotid artery and endothelial dysfunction in severely obese children: a prospective study Lancet 2001 358 9291 1400 1404 10.1016/S0140-6736(01)06525-4 11705484

[B40] Sakuragi S Influence of adiposity and physical activity on arterial stiffness in healthy children: the lifestyle of our kids study Hypertension 2009 53 4 611 616 10.1161/HYPERTENSIONAHA.108.123364 19273744

[B41] Wang JJ Retinal vessel diameter and cardiovascular mortality: pooled data analysis from two older populations Eur Heart J 2007 28 16 1984 1992 10.1093/eurheartj/ehm221 17626032

[B42] Wong TY Retinal arteriolar narrowing and risk of coronary heart disease in men and women. The Atherosclerosis Risk in Communities Study JAMA 2002 287 9 1153 1159 10.1001/jama.287.9.1153 11879113

[B43] Cheung N BMI and retinal vascular caliber in children Obesity (Silver Spring) 2007 15 1 209 215 10.1038/oby.2007.576 17228049

[B44] Mitchell P Blood pressure and retinal arteriolar narrowing in children Hypertension 2007 49 5 1156 1162 10.1161/HYPERTENSIONAHA.106.085910 17372033

[B45] AAP American Academy of Pediatrics: Children, adolescents, and television Pediatrics 2001 107 2 423 426 11158483 10.1542/peds.107.2.423

[B46] Aktas A The effects of television food advertisement on children's food purchasing requests Pediatrics International 2006 48 138 145 10.1111/j.1442-200X.2006.02180.x 16635172

[B47] Ludwig DS Gortmaker SL Programming obesity in childhood Lancet 2004 364 9430 226 227 10.1016/S0140-6736(04)16688-9 15262082

[B48] Epstein LH A randomized trial of the effects of reducing television viewing and computer use on body mass index in young children Arch Pediatr Adolesc Med 2008 162 3 239 245 10.1001/archpediatrics.2007.45 18316661 PMC2291289

[B49] Robinson TN Reducing children's television viewing to prevent obesity: a randomized controlled trial JAMA 1999 282 16 1561 1567 10.1001/jama.282.16.1561 10546696

[B50] Hancox RJ Milne BJ Poulton R Association of television viewing during childhood with poor educational achievement Arch Pediatr Adolesc Med 2005 159 7 614 618 10.1001/archpedi.159.7.614 15996992

[B51] Winnail SD Relationship between physical activity level and cigarette, smokeless tobacco, and marijuana use among public high school adolescents J Sch Health 1995 65 10 438 442 10.1111/j.1746-1561.1995.tb08209.x 8789710

[B52] Ells LJ Prevention of childhood obesity Best Pract Res Clin Endocrinol Metab 2005 19 3 441 454 10.1016/j.beem.2005.04.008 16150385

[B53] Harris KC Effect of school-based physical activity interventions on body mass index in children: a meta-analysis CMAJ 2009 180 7 719 726 19332753 10.1503/cmaj.080966 PMC2659836

[B54] Kropski JA Keckley PH Jensen GL School-based obesity prevention programs: an evidence-based review Obesity (Silver Spring) 2008 16 5 1009 1018 10.1038/oby.2008.29 18356849

[B55] Zenzen W Kridli S Integrative review of school-based childhood obesity prevention programs J Pediatr Health Care 2009 23 4 242 258 10.1016/j.pedhc.2008.04.008 19559992

[B56] Kriemler S Effect of school based physical activity programme (KISS) on fitness and adiposity in primary schoolchildren: cluster randomised controlled trial BMJ 2010 340 c785 10.1136/bmj.c785 20179126 PMC2827713

[B57] Donner A Klar N Design and Analysis of Cluster Randomization Trials in Health Research 2000 Arnold

[B58] Liang KY Zeger S Longitudinal data analysis using generalized linear models Biometrika 1986 73 13 22 10.1093/biomet/73.1.13

[B59] Sharples K Breslow N Regression Analysis of Correlated Binary Data: Some Small Sample Results for the Estimating Equation Approach Journal of Statistical Computation and Simulation 1992 42 1 20

[B60] Donner A Klar N Statistical Considerations in the Design and Analysis of Community Intervention Trials Journal of Clinical Epidemiology 1996 49 435 439 10.1016/0895-4356(95)00511-0 8621994

[B61] Bandura A Health promotion by social cognitive means Health Educ Behav 2004 31 2 143 164 10.1177/1090198104263660 15090118

[B62] Cole TJ The LMS method for constructing normalized growth standards Eur J Clin Nutr 1990 44 1 45 60 2354692

[B63] Kromeyer-Hauschild K Perzentile für den Body Mass Index für das Kindes- und Jugendalter unter Heranziehung verschiedener deutscher Stichproben Monatsschrift Kinderheilkunde 2001 149 807 818 10.1007/s001120170107

[B64] The fourth report on the diagnosis, evaluation, and treatment of high blood pressure in children and adolescents Pediatrics 2004 114 2 Suppl 4th Report 555 576 15286277

[B65] Chen CH Estimation of central aortic pressure waveform by mathematical transformation of radial tonometry pressure. Validation of generalized transfer function Circulation 1997 95 7 1827 1836 9107170 10.1161/01.cir.95.7.1827

[B66] Hubbard LD Methods for evaluation of retinal microvascular abnormalities associated with hypertension/sclerosis in the Atherosclerosis Risk in Communities Study Ophthalmology 1999 106 12 2269 2280 10.1016/S0161-6420(99)90525-0 10599656

[B67] Rusch H Irrgang W Münchner Fitnesstest Haltung und Bewegung 1994 14 1 4 11

[B68] Prochaska JJ Sallis JF Long B A physical activity screening measure for use with adolescents in primary care Arch Pediatr Adolesc Med 2001 155 5 554 559 11343497 10.1001/archpedi.155.5.554

[B69] Bös K Worth A Opper E Oberger J Woll A Motorik-Modul Motorische Leistungsfähigkeit und körperlich-sportliche Aktivität von Kindern und Jugendlichen in Deutschland. The motoric-module: motor performance ability and physical activity of children and adolescents in Germany 2009 1 Nomos-Verlag, Baden-Baden 354 361

[B70] Mensink G Kleiser C Richter A Lebensmittelverzehr bei Kindern und Jugendlichen in Deutschland - Ergebnisse des Kinder- und Jugendgesundheitssurveys (KiGGS) Bundesgesundheitsblatt - Gesundheitsforschung - Gesundheitsschutz 2007 50 5/6 609 623 17514445 10.1007/s00103-007-0222-x

[B71] Ravens-Sieberer U Bullinger M Assessing health-related quality of life in chronically ill children with the German KINDL: first psychometric and content analytical results Qual Life Res 1998 7 5 399 407 9691720 10.1023/a:1008853819715

[B72] Brambilla P Pozzobon G Pietrobelli A Physical activity as the main therapeutic tool for metabolic syndrome in childhood Int J Obes (Lond) 2010 in press 10.1038/ijo.2010.255 21139560

[B73] Tudor-Locke C Omission of active commuting to school and the prevalence of children's health-related physical activity levels: the Russian Longitudinal Monitoring Study Child Care Health Dev 2002 28 6 507 512 10.1046/j.1365-2214.2002.00295.x 12568480

